# Histological and Immunohistological Alterations in Carrot Roots and Leaves Under Salt Stress

**DOI:** 10.3390/ijms262412027

**Published:** 2025-12-14

**Authors:** Ewa Kurczyńska, Katarzyna Sala-Cholewa, Kamila Godel-Jędrychowska, Kamil Szymonik, Magdalena Klimek-Chodacka, Rafal Baranski

**Affiliations:** 1 Institute of Biology, Biotechnology and Environmental Protection, Faculty of Natural Sciences, University of Silesia in Katowice, Jagiellońska 28, 40-032 Katowice, Poland; ewa.kurczynska@us.edu.pl (E.K.); kamila.godel@gmail.com (K.G.-J.); 2 Department of Plant Biology and Biotechnology, Faculty of Biotechnology and Horticulture, University of Agriculture in Krakow, Al Mickiewicza 21, 31-120 Krakow, Poland; kamil.szymonik@urk.edu.pl (K.S.); magdalena.klimek-chodacka@urk.edu.pl (M.K.-C.)

**Keywords:** anatomy, arabinogalactan proteins, cell wall components, *Daucus carota*, histochemistry, pectins, salt stress tolerance, soil salinity, starch

## Abstract

Salt stress significantly reduces plant growth and yield, which has led to extensive research on the mechanisms underlying plant salinity tolerance. Carrot (*Daucus carota* ssp. *sativus*) is a glycophyte highly sensitive to soil salinity. We investigated root and leaf anatomical, histological, and immunohistological alterations in two carrot accessions, previously identified as salt-sensitive (DH1) and salt-tolerant (DLBA), growing under control and salt stress conditions. The results demonstrate that the salt-tolerant DLBA growing under control conditions has trichome-rich leaves, high starch reserves and a hydraulically safer root xylem. Under salt stress, DLBA maintains mesophyll integrity, and increases the number of vessels and deposition of highly esterified pectins, hemicelluloses and spatially regulated AGPs in cell walls. In contrast, DH1 develops thinner, trichome-free leaves, and roots almost free of starch with fewer cambial cells and vessels. Salt stress induces overexpansion of palisade parenchyma, excess starch accumulation, loss of arabinan epitopes, disappearance of extensins in vascular bundles, and changes in hemicellulose and AGP distribution. These findings indicate that salt tolerance of DLBA plants results from the combination of constitutive anatomical characteristics and adaptive responses that together support tissue hydration, wall elasticity and stable water transport when plants are growing in saline soil.

## 1. Introduction

Soil salinity is an escalating global challenge, currently affecting over 20% of arable land and approximately 33% of irrigated areas worldwide. The main drivers of salinization include desertification linked to climate change, seawater intrusion, and intensive irrigation practices in agricultural systems. Elevated salinity restricts plant growth and reduces crop yields, posing a major threat to global food security [1,2].

Plant responses to salinity stress typically involve two partially overlapping phases. In the osmotic phase, reduced soil water potential limits water uptake, leading to turgor loss and stomatal closure, which in turn decreases internal CO_2_ concentration. The subsequent ionic phase results from the excessive accumulation of sodium and chloride ions, disrupting ionic homeostasis and interfering with potassium-dependent metabolic processes. These changes induce oxidative stress and disturb redox equilibrium. The combined osmotic, ionic, and oxidative imbalances damage chloroplasts, decrease photosynthetic efficiency, and impair assimilate distribution within the plant [3].

Plants have evolved a range of anatomical and physiological strategies to tolerate saline conditions. Salt-tolerant species often develop thicker leaf blades and a modified palisade-to-spongy parenchyma ratio. Enlarged intercellular spaces in the spongy parenchyma enhance CO_2_ diffusion and help maintain water balance [4,5]. The outer tissues, including the epidermis (with cuticle) and periderm, are typically more developed, supporting ion influx control and homeostasis. A thicker root periderm and a modified endoderm limit the apoplastic flow of Na^+^ and Cl^−^ while enhancing K^+^ retention [6]. Alterations in xylem structure also facilitate efficient water transport under saline stress [4,7].

Initial responses to salinity occur at the cellular level, particularly in membranes and the cell wall. The primary cell wall is a dynamic structure capable of rapid adjustment to osmotic and ionic fluctuations [8,9]. Modifications in pectin content and methylation, mediated by pectin methylesterases and their inhibitors, influence wall charge, Ca^2+^ cross-linking capacity, and water-binding properties [9,10]. Changes in hemicellulose composition, particularly xyloglucans, affect wall stiffness and promote anisotropic cell growth under reduced turgor. Cell wall glycoproteins also play key roles in stress adaptation: arabinogalactan proteins (AGPs) can bind Ca^2+^ and scavenge reactive oxygen species (ROS), contributing to redox balance and cell expansion, while extensins reinforce and repair the wall under oxidative conditions [11]. Collectively, these molecular and structural adjustments help stabilize wall rigidity, osmotic balance, ion transport, and ROS homeostasis.

Carrot (*Daucus carota* ssp. *sativus*) is a glycophytic crop sensitive to soil salinity. Root yield declines even at low soil electrical conductivity [12], although some landraces exhibit greater tolerance and can grow under saline conditions [13]. Our previous studies demonstrated pronounced genotypic differences between the salt-sensitive DH1 line and the salt-tolerant DLBA accession. Compared with DH1, DLBA exhibited lower biomass reduction under salt stress, improved ion regulation, and a more stable K^+^/Na^+^ ratio [14]. It also showed higher peroxidase activity, a more efficient ascorbate–glutathione cycle, and greater proline accumulation, indicating enhanced antioxidant defense and osmoprotection [15].

Comparative analyses of carrot miRNAomes and degradomes under salt stress revealed clear differences in miRNA expression and target regulation between the two genotypes [16]. The identified regulatory genes were involved in signaling, antioxidant defense, and particularly in cell wall remodeling. In DLBA, *AGP9L* (encoding arabinogalactan protein) was upregulated, while *XXT1* (encoding xyloglucan xylosyltransferase) was downregulated, suggesting that salt stress induces modifications in cell wall composition, characterized by increased AGP content and decreased xyloglucan levels [16]. These molecular findings highlight the need for detailed anatomical and histochemical analyses of carrot responses to salinity. Such studies can link cell wall composition and tissue organization to the previously identified physiological and molecular mechanisms in sensitive and tolerant genotypes. Therefore, the present study aimed to examine histological alterations and the distribution of key cell wall components in the salt-sensitive DH1 and salt-tolerant DLBA carrot accessions. The results provide new insights into the anatomical and structural traits underlying salinity tolerance in carrot.

## 2. Results

### 2.1. Root Histology

**Figure 1:**
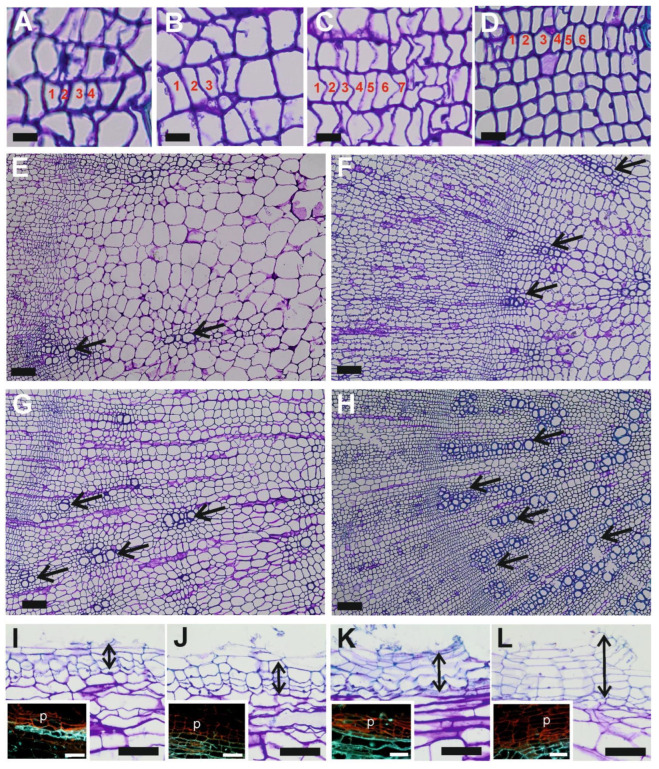
Representative images of the cambial zone in DH1 roots under control (**A**) and NaCl-treated (**B**) conditions, and in DLBA roots under control (**C**) and salt stress (**D**) conditions (numbers indicate cambial cells per radial row; scale bar = 20 µm). Representative root cross-sections of DH1 under control (**E**) and salt stress (**F**), and DLBA under control (**G**) and salt stress (**H**) (arrows indicate selected vessels; scale bar = 100 µm). Representative images of the peridermal layer in DH1 control (**I**) and NaCl-treated (**J**) roots, and DLBA control (**K**) and NaCl-treated (**L**) roots (scale bars = 50 µm). Insets show autofluorescence of cork cell walls (vector indicates peridermal layer; p, periderm; scale bars = 25 µm).

The number of cambial cells varied significantly depending on the accession and treatment (Table 1, Figure 1A–D). In DH1 roots, the mean number of cambial cells per radial row was 4.1 under control conditions and decreased by 48% under salt stress (*p* < 0.001). In DLBA roots, the mean number was higher (6.5; *p* < 0.001 compared with DH1) and also declined under salt stress (*p* < 0.001), but only by 18%. Despite this reduction, the number of cambial cells in salt-stressed DLBA roots remained approximately twice that in stressed DH1 roots.

In both accessions, the mean vessel diameter (d) did not differ under control conditions (*p* = 0.147) and decreased by about 20% under salt stress (*p* < 0.001). Thus, vessel size was comparable between accessions, but consistently smaller under salinity.

The proportion of radial rows containing vessels (R) was similar between accessions (*p* = 0.540) and was not significantly affected by salt stress (*p* = 0.236), although a slight increasing trend was observed in DLBA (Table 1, Figure 1E–H). In contrast, the interaction between accession and treatment for the proportion of vessels among the total number of cells per radial row (V) was highly significant (*p* < 0.001). This proportion was three times higher in DLBA under salt stress than under control conditions (*p* < 0.001), whereas no significant change was observed in DH1 (*p* = 0.075).

To assess the functional consequences of the anatomical changes, we combined R, V and d hydraulically relevant anatomical traits to calculate a relative index of vessel lumen area (R·V·d^2^). In DH1, the index decreased from 89 µm^2^ to 32 µm^2^ under salt stress (a 65% reduction), whereas in DLBA it increased more than 3-fold relative to the control, rising from 42 µm^2^ to 133 µm^2^. A consistent but more pronounced pattern was obtained for the relative hydraulic conductance index (R·V·d^4^), derived from the Hagen–Poiseuille relationship. In DH1, theoretical conductance decreased 4.3-fold under salt stress compared with the control (88,755 µm^4^ vs. 20,507 µm^4^), corresponding to a relative ratio of 1.00:0.23. In DLBA, it doubled from 49,744 µm^4^ to 99,345 µm^4^ exceeding the DH1 control level (relative ratio to the DH1 control set to 1.00:0.56 and 1.12).

Differences in periderm development were also observed. The cork layer was assessed visually, as accurate quantification of cork cells was not possible due to frequent peeling of the surface layers. Regardless of accession, the lowest number of cork cells per radial row was observed in control roots (Figure 1). Salt stress did not affect periderm thickness in DH1, but it increased periderm thickness in DLBA, resulting in the thickest periderm in NaCl-treated DLBA roots (Figure 1I–L).

**Figure 2:**
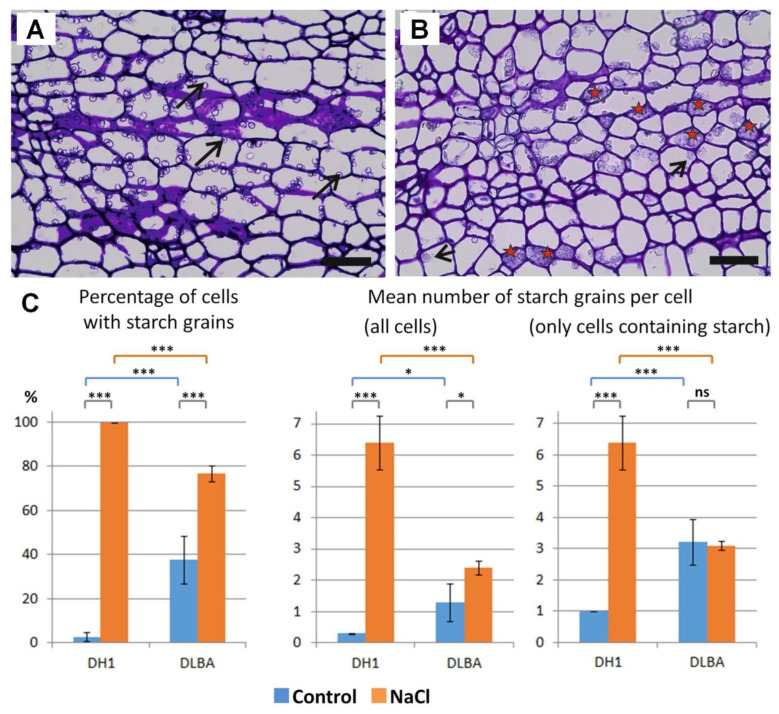
Presence and number of starch grains in cortical parenchyma cells of DH1 (**A**) and DLBA (**B**) NaCl-treated roots (arrows indicate starch grains; red stars mark cells filled with starch; scale bars = 100 µm). (**C**) Bar charts show the percentage of cells containing starch grains and the mean number of grains per cell, calculated either for all cells or only for starch-containing cells. Three replicates were analyzed, each including 300 cells. Whiskers represent standard deviations. Asterisks indicate significant differences between means according to the Neuman–Keuls multiple comparison test (*** *p* < 0.001; * *p* < 0.05; ns, not significant).

The presence and abundance of starch grains were examined in cortical parenchyma cells (Figure 2). In DH1 control roots, starch grains were nearly absent, whereas in salt-treated roots, all cells contained starch, with a mean of 6.4 grains per cell. In contrast, DLBA control roots had starch grains in 37.7% of cells, with an average of 1.3 grains per cell. Under salt stress, the proportion of starch-containing cells in DLBA increased to 77.0%, and the mean number of grains per cell nearly doubled to 2.4. Differences between accessions and treatments were highly significant (all *p* < 0.001). When only starch-containing cells were considered, the mean number of grains per cell increased significantly in DH1 under salt stress but remained unchanged in DLBA (*p* < 0.001).

### 2.2. Root Immunohistology

**Figure 3:**
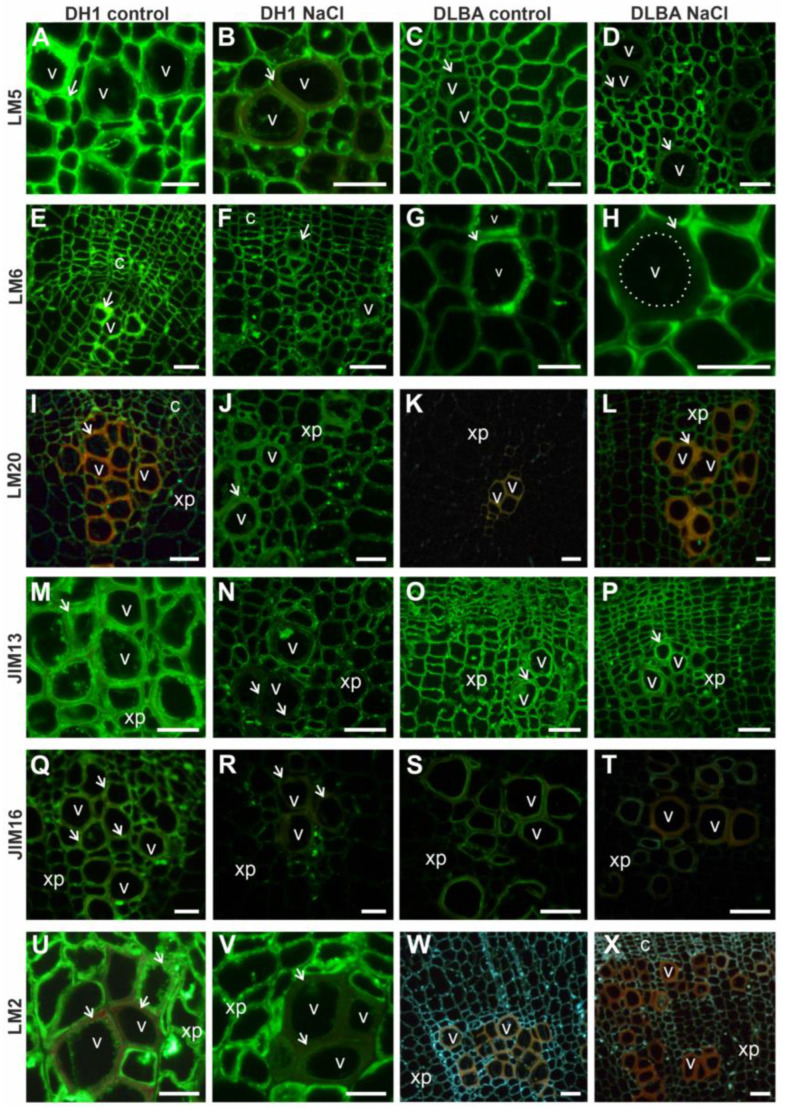
Distribution of the analyzed pectic (**A**–**L**) and AGP (**M**–**X**) epitopes in the cells of DH1 and DLBA roots under control and salt stress conditions. In panels (**W**,**X**), the red color of the vessel walls and the blue color of other cell walls result from the use of the auto-white option, which automatically adjusts white balance, exposure, and contrast in the microscope imaging system, ensuring accurate and consistent color rendering without manual input. c—cambium; v—vessel; xp—xylem parenchyma; arrows indicate the vessel cell wall. In panel (**H**), the white dashed line marks the inner boundary of the vessel cell wall; scale bars = 50 µm.

All analyzed epitopes were localized in cell walls when detected. Among the pectic epitopes, the LM5 antibody recognized a component that was abundant in all tissues of control roots in both DH1 and DLBA (Figure 3A,C), whereas under salt stress, it was present in the cell walls of most cells except of mature vessels (Figure 3B,D). A similar pattern was observed for the LM6 antibody (Figure 3E–H).

In control DH1 roots, the LM20 epitope was present in all tissues except vessel walls, while in NaCl-treated roots, it was also detected in vessel walls (Figure 3I,J). In control DLBA roots, LM20 was not detected in any tissue, whereas under salt stress, it appeared in all tissues except the walls of mature vessels (Figure 3K,L). The LM19 epitope (unesterified or low methyl-esterified homogalacturonan) was not detected in any accession or treatment.

**Figure 4:**
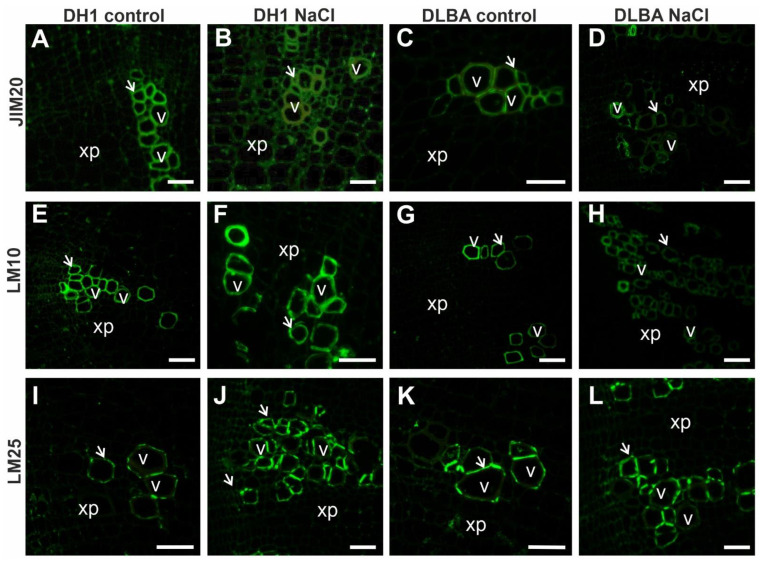
Distribution of the analyzed extension (**A**–**D**), xylan (**E**–**H**) and xyloglucan (**I**–**L**) epitopes detected in the cells of DH1 and DLBA roots under control and salt stress conditions (v—vessel; xp—xylem parenchyma; arrows point to vessel cell wall; scale bars = 50 µm).

**Figure 5:**
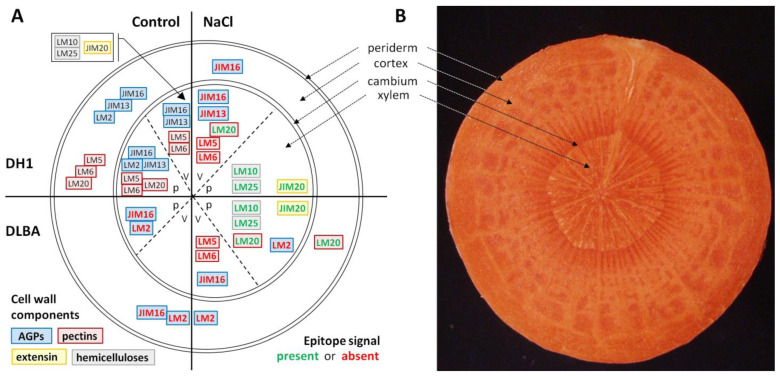
Diagram (**A**) with a reference to a root cross-section (**B**) illustrating the distribution of the analyzed epitopes (black fonts) in the DH1 root under control conditions, the differences in their distribution in DLBA control relative to DH1, and the changes observed in both accessions under salt stress. Green and red fonts indicate the presence and absence of the epitope, respectively (only differences between accessions and treatments are shown). Cell wall components of the same group are outlined in specific colors: blue—arabinogalactan proteins, red—pectins, yellow—extensin, and grey—hemicelluloses. Abbreviations: v—xylem vessels; p—xylem parenchyma.

The AGP epitope recognized by JIM13 was present in all tissues of DH1 control roots, but absent from vessel walls in NaCl-treated DH1 roots (Figure 3M,N). In DLBA roots, this epitope was abundant in all tissues regardless of treatment (Figure 3O,P). The JIM16 epitope was present in various tissues, including vessel walls, in DH1 control roots (Figure 3Q), but under salt stress, it was limited to some xylem parenchyma cells near the vessels (Figure 3R). In DLBA control roots, JIM16 was detected only in mature vessel walls (Figure 3S), while under salt stress, it was restricted to the walls of some vessels and accompanying xylem parenchyma cells (Figure 3T). The LM2 epitope was abundant in all DH1 root tissues except vessel walls, independent of treatment, with this pattern particularly evident under salt stress (Figure 3U,V). In DLBA roots, LM2 was not detected in any tissue (Figure 3W,X), where only tissue autofluorescence was observed. Epitopes of extensin (JIM20), xylan (LM10), and xyloglucan (LM25) were primarily detected in vessel walls, independent of accession (Figure 4). In JIM20-labeled sections, the signal was restricted to vessel walls in control roots of both accessions (Figure 4A,C), but in NaCl-treated roots, it also appeared in some xylem parenchyma cells (Figure 4B,D). Under salt stress, xylan (LM10) and xyloglucan (LM25) epitopes were additionally observed in the walls of xylem parenchyma cells in DLBA, although the fluorescence signal was weak (Figure 4E–L). Epitopes showing differential tissue distribution between accessions and treatments are summarized in a schematic diagram (Figure 5).

### 2.3. Leaf Histology

**Figure 6:**
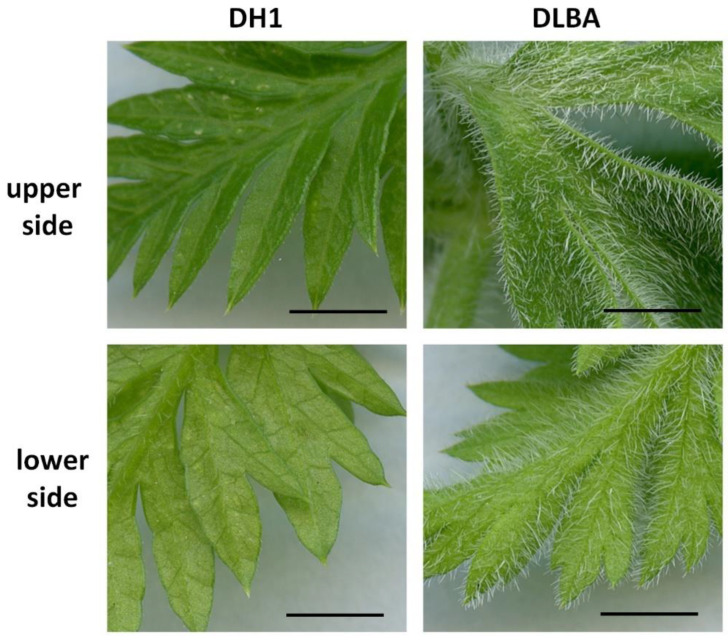
Trichomes on the upper and lower surfaces of the DH1 and DLBA leaves. The DLBA leaves are densely covered with trichomes on both surfaces, whereas the DH1 leaves possess only a few trichomes and have smoother surfaces (scale bars = 3 mm).

The DLBA accession was characterized by a high density of trichomes on both the upper (adaxial side) and lower (abaxial side) surfaces of the leaf blade as well as on the petioles (Figure 6). In the DLBA plants exposed to salt stress, the number of trichomes on the upper leaf surface (33.6 per mm^2^) decreased by about 40% (*p* < 0.001), while on the lower surface it increased from 15.1 per mm^2^ by 53% (*p* < 0.001) compared to the control and reached 23.2 per mm^2^ (Table 2). In the DH1 plants, trichomes occurred only rarely, regardless of treatment, and the blades were more deeply lobed and visibly thinner. The DLBA blades were 306 µm thick and thus 16% thicker than those of DH1 (264 µm) (*p* = 0.025) (Table 2). Under salt stress, thickness increased by 16% in DLBA (*p* = 0.003) and by 9% in DH1, which was found non-significant. The increase resulted from the thickening of the upper epidermis and the spongy parenchyma, and in case of DH1, additionally by a reduction in the palisade parenchyma. The thickness of the upper and lower epidermis was similar in DH1 and DLBA (*p* = 0.856 and *p* = 0.796, respectively). Changes in the upper epidermis under salt stress (*p* < 0.001) were more pronounced in DH1 (37% increase) than in DLBA (16% increase). The change in lower epidermis thickness was not significant in any accession (*p* = 0.392).

**Figure 7:**
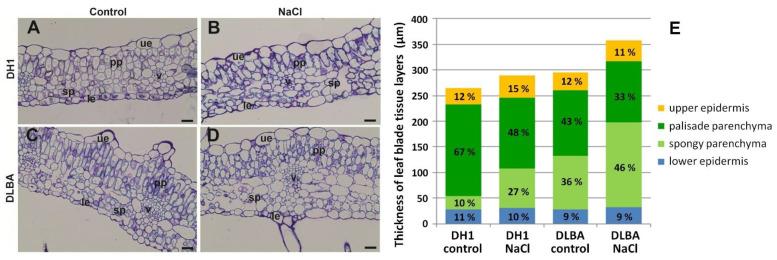
Transverse sections of the leaves of the DH1 and DLBA accessions under the control (**A**,**C**) and salt stress (**B**,**D**) conditions. Stacked bar chart (**E**) showing leaf blade thickness divided into individual tissue layers. Percentage values indicate the proportion of each tissue layer in the total leaf thickness (ue—upper epidermis; le—lower epidermis; pp—palisade parenchyma; sp—spongy parenchyma; v—vascular bundle; scale bars = 50 µm).

The palisade parenchyma layer in the DLBA leaf blade was 28% thinner than in the DH1 leaf (*p* < 0.001) and remained unchanged under salt stress (*p* = 0.472). In contrast, in DH1, its thickness decreased (*p* = 0.002) by 22% (from 178 µm to 138 µm), which was similar to that in DLBA (120 µm) (*p* = 0.196) (Figure 7). The spongy parenchyma in DLBA was four times thicker than in DH1 (*p* < 0.001) and increased in thickness by 60 µm (i.e., by 56%) under salt stress (*p* = 0.002), whereas in DH1 it increased by 50 µm and was almost tripled (2.9-fold increase) (*p* = 0.008). The spongy parenchyma of DLBA was also more loosely arranged in comparison to DH1, with 80% larger intercellular spaces (measured as the area of intercellular spaces in the leaf cross-section) in the control (*p* = 0.002) and 2.5-fold larger under salt stress (*p* < 0.001). Consequently, the ratio of palisade to spongy parenchyma thickness in DH1 decreased from 6.7 to 1.8. In DLBA, the thickness of both layers was similar under control conditions (ratio = 1.2), whereas under salt stress the palisade parenchyma became thinner than the spongy parenchyma (ratio = 0.7).

### 2.4. Leaf Immunohistology

**Figure 8:**
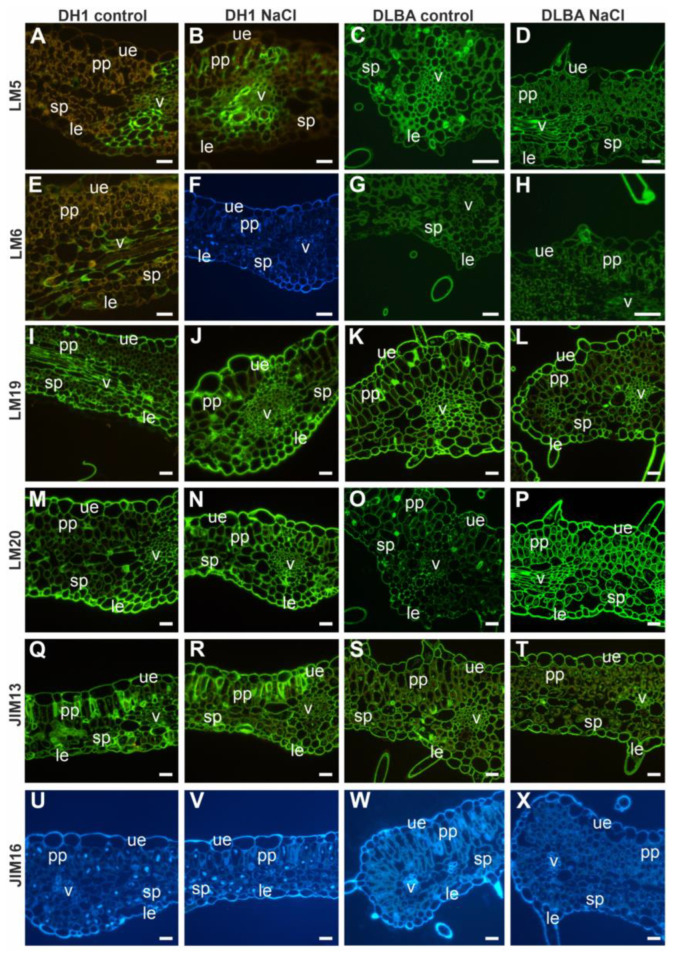
Distribution of the analyzed pectic (**A**–**P**) and AGP (**Q**–**X**) epitopes in the cells of the DH1 and DLBA leaves under the control and salt stress conditions (the blue images: (**F**,**U**–**X**) represent leaf cross-sections visualized under UV light after applying FB28 to stain cellulose; they are shown instead of the sections immunolabeled for individual epitopes, where the images appear black due to the absence of these epitopes). Abbreviations: ue—upper epidermis; le—lower epidermis; pp—palisade parenchyma; sp—spongy parenchyma; v—vascular bundle. Scale bars = 50 µm.

**Figure 9:**
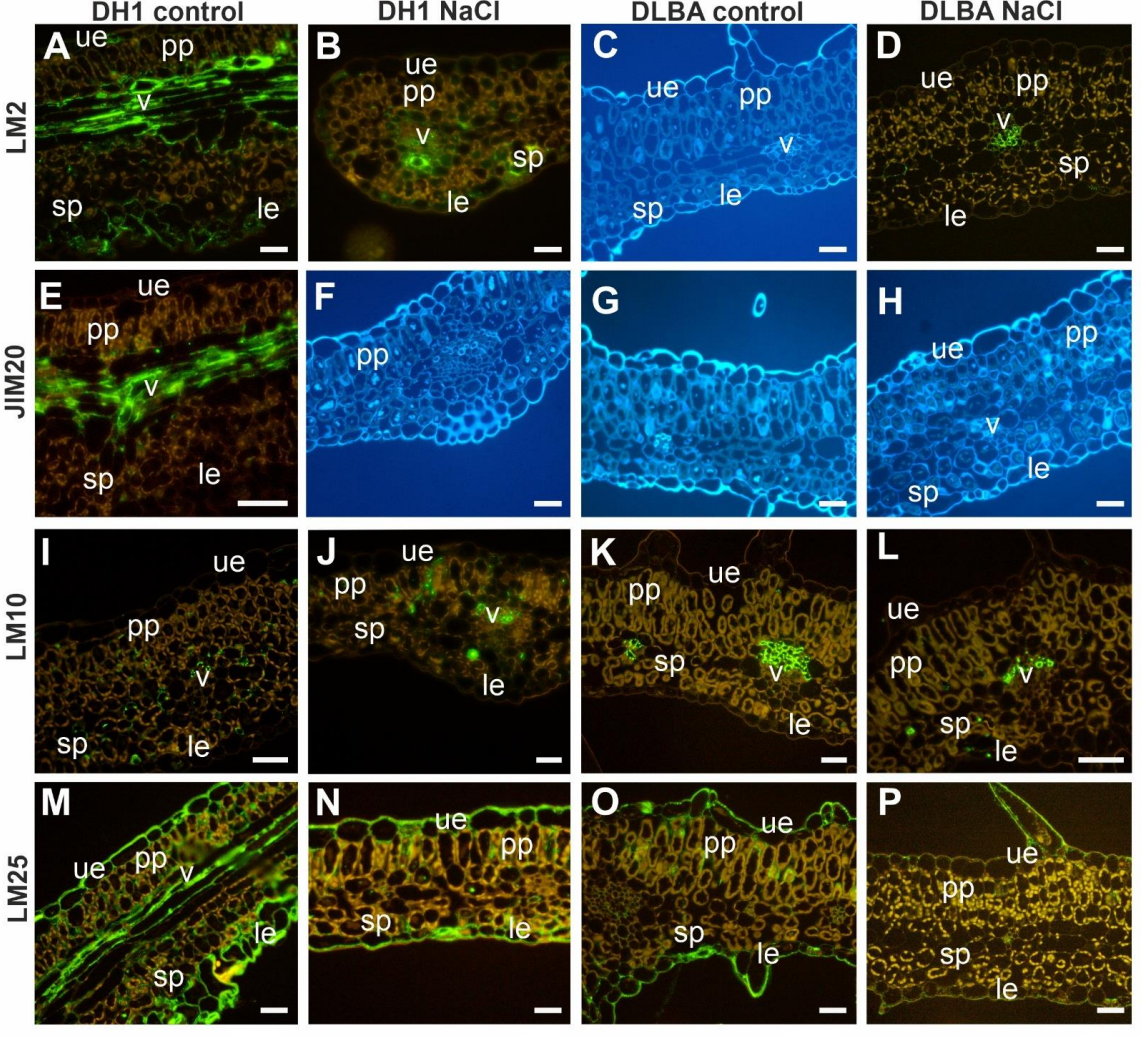
Distribution of the analyzed AGP (**A**–**D**), extension (**E**–**H**), xylan (**I**–**L**) and xyloglucan (**M**–**P**) epitopes in the cells of DH1 and DLBA leaves under control and salt stress conditions. Blue images (**C**,**F**–**H**) represent leaf cross-sections visualized under UV light after staining cellulose with FB28. These are shown instead of the sections immunolabeled for individual epitopes, where the images appear black due to the absence of labeling. Abbreviations: ue—upper epidermis; le—lower epidermis; pp—palisade parenchyma; sp—spongy parenchyma; v—vascular bundle. Scale bars = 50 µm.

The pectic epitope recognized by the LM5 antibody in control DH1 leaves was localized in the cell walls of the vascular bundle and bundle sheath, whereas in salt-stressed plants, it was additionally present in the cell walls of palisade parenchyma cells (Figure 8A,B). In DLBA leaves, LM5 was abundant in all cell walls regardless of treatment (Figure 8C,D). The LM6 epitope in control DH1 leaves was detected only in vascular bundle walls and some lower epidermal cells, but it was absent under salt stress (Figure 8E,F). In contrast, LM6 was present in all leaf tissues of DLBA plants under both conditions (Figure 8G,H). The LM19 epitope was detected in all leaf tissues in both accessions, independent of treatment (Figure 8I–L), while LM20 was more abundant in NaCl-treated DH1 and DLBA leaves than in controls (visual estimation) (Figure 8M–P). The AGP epitope recognized by JIM13 was present in all leaf tissues (Figure 8Q–T), whereas JIM16 was not detected in any tissue, irrespective of accession or treatment (Figure 8U–X). In DH1 leaves, LM2 was localized in the vascular bundle, in some upper and lower epidermal cells, and in certain spongy parenchyma cells under both control and salt stress (Figure 9A,B). In DLBA leaves, LM2 was absent in control plants but appeared in the vascular bundles under salt stress (Figure 9C,D). The extensin epitope JIM20 was restricted to vascular bundles in control DH1 leaves (Figure 9E–H). The xylan epitope LM10 was mainly observed in vascular bundles regardless of accession or treatment, with additional signals in some palisade and spongy parenchyma cells of DH1 leaves (Figure 9I–L). The xyloglucan epitope LM25 was detected in upper and lower epidermal cells in all leaves, and in the vascular bundles of control DH1 leaves (Figure 9M–P).

**Figure 10:**
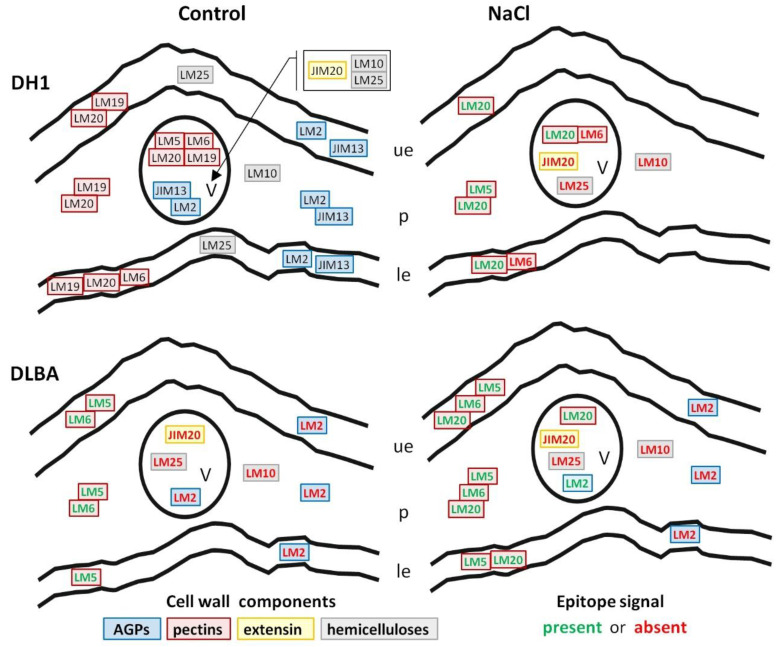
Diagram illustrating the distribution of the analyzed epitopes (black fonts) in the DH1 leaf under control conditions, the differences in their distribution in the DLBA control relative to DH1, and the changes observed in both accessions under NaCl stress. Green and red fonts indicate the presence and absence of the epitope, respectively (only differences between accessions and treatments are shown). Cell wall components of the same group are outlined in specific colours: blue—arabinogalactan proteins, red—pectins, yellow—extensin, and grey—hemicelluloses. Abbreviations: v—vascular bundle; ue—upper epidermis; p—mesophyll parenchyma; le—lower epidermis.

In summary, DH1 leaves under salt stress lacked arabinan, extensin, and xyloglucan epitopes in vascular bundles, whereas in DLBA leaves under salt stress, the LM2 AGP epitope appeared in vascular bundles. The main differences between DH1 and DLBA were the absence of arabinans and galactans in the epidermis and parenchyma of DH1 leaves (Figure 10).

## 3. Discussion

Plant cell wall structure, composition and metabolism are dynamically regulated in response to a variety of biotic and abiotic stresses including salinity. Using state-of-the-art histological and immunohistological tools we examined the histological changes in roots and leaves as well as the presence and distribution of chemical constituents of the walls of two different *Daucus carota* accessions, DH1 and DLBA, exposed to salt stress in comparison to the control plants.

### 3.1. Cambium and Vessels in Roots

Research on the impact of salt stress on plants focuses mainly on physiological and molecular responses, whereas detailed analyses of cambial activity remain limited, despite the cambium being the meristem responsible for producing xylem and phloem cells. A reduction in the cambial zone under salinity has been described for some herbaceous and woody plants, e.g., *Raphanus sativus* [17], *Glycine max* [18], *Capsicum annuum* [19], *Populus x canescens* [20], *Barringtonia racemosa* [21]. The presented here results indicate that salinity reduces the number of cells in the root cambial zone of both *Daucus carota* accessions, indicating decreased meristematic activity and consequently reduced production of secondary xylem and phloem. It is postulated that the adverse effect of salinity on the cambium results from the fact that osmotic stress induced by Na^+^ reduces the cell division activity [18,22]. The higher number of cambial cells observed in DLBA compared with DH1 may explain a greater capacity of DLBA for sustaining secondary growth and lower reduction in biomass under salt stress conditions as shown previously [14].

At the cellular level, salt stress altered xylem differentiation which resulted in an increased number of vessels and reduced vessel diameter, which was particularly visible in DLBA roots. Similar responses have been described in *Solanum lycopersicum* [23], *Setaria italica* [24] and *Tamarix ramosissima* [25]. It was postulated that an increased number of tracheary elements in roots under salt stress may enhance the cell-to-cell pathway for water transport, which would impart greater selectivity and reduced ion uptake, and compensate for diminished bulk flow of water and solutes along the apoplastic pathway [23]. The strong increase in vessel density in DLBA resembles patterns observed in salt-tolerant species such as *Rhizophora mucronata*, where high vessel density has been interpreted as a safety strategy [26].

The DH1 reaction was different, the density of vessels was significantly reduced under salt stress. Similar changes were reported in other species as a reaction to salt stress causing limited growth, e.g., *Zea mays* [27], *Carthamus tinctorius* [28] and *Atriplex semibaccata* [29].

These anatomical alterations were further quantified using relative hydraulic indices. Both the estimated vessel lumen area (R · V · d^2^) and the theoretical hydraulic conductance (R · V · d^4^), derived from the Hagen–Poiseuille relationship, decreased markedly in DH1 but increased in DLBA under salt stress. Because hydraulic conductance depends on the fourth power of conduit radius, even moderate reductions in vessel diameter strongly limit flow, whereas increased vessel frequency can compensate for such losses [30]. Although smaller vessels reduce the conductivity of individual conduits, they enhance capillary forces and resistance to cavitation, hence hydraulic safety [31,32]. Thus higher vessel density observed in DLBA can increase whole-tissue conductance under salt stress conditions.

These findings also align with previously reported physiological differences between the two accessions [15]. Although leaf relative water content did not differ significantly between DH1 and DLBA, the latter showed lower oxidative damage and a stronger activation of antioxidant systems under salinity. This indicates that DLBA experiences less severe stress despite having a similar overall water status. A likely explanation is that its higher vessel density helps maintain a more stable water flow through the tissues, limiting hydration fluctuations that can trigger oxidative damage [33]. In contrast, the reduction in vessel number and diameter in DH1 probably disrupts the stability of water transport, contributing to higher oxidative stress despite similar relative water content. Thus, the two accessions exhibit fundamentally different hydraulic strategies. The salt-tolerant DLBA accession develops a hydraulically safer and more redundant root xylem while in the salt-sensitive DH1, salt stress induces a decline in both hydraulic efficiency and safety.

### 3.2. Storage Sugars in Roots

Salt stress changes carbon allocation very strongly, and accumulation of starch is often observed in plants growing in saline soil [34,35]. Osmoprotection depends mainly on soluble sugars and other compatible solutes [36]. Starch is a storage form with low osmotic activity and it can be mobilized to produce soluble carbohydrates for osmotic adjustment. In our study, both accessions increased the amount of starch in cortical parenchyma under salt stress, but the scale and pattern of these changes were different between the accessions. DH1, which has almost no starch in control conditions, represents typical orange edible carrots that usually contain very little starch and depend mainly on soluble sugars [37]. The strong induction of starch synthesis and its large accumulation under salinity therefore shows a major shift from its normal carbohydrate balance. The large redirection of carbon into starch in DH1 suggests that the accession has lower availability of soluble osmolytes and that its sugar homeostasis becomes disturbed. This idea agrees with earlier results showing that soluble sugars in DH1 leaves decrease under salt stress [15]. Such big starch accumulation is also connected with low sink activity or problems with starch remobilization, which are known to limit metabolic support during stress [38]. In contrast, DLBA has a relatively large starch reserve already in control conditions, and this pool increases only moderately after salt treatment. This stable carbohydrate status means that DLBA does not need to change carbon allocation so drastically when stress appears. The constitutive starch reserve can be mobilized when photosynthesis becomes limited, giving a constant source of hexoses and sucrose and helping to keep osmotic and metabolic balance. Altogether, these results suggest that DLBA is better prepared for salt stress because it keeps a more stable sugar management in both control and stress conditions, while DH1 shows a clear disturbance of carbon allocation when salinity occurs. We hypothesize that DH1 accumulates large amounts of starch as a survival strategy, storing carbon for future use when growth conditions improve. Biennial plants such as carrots need carbon reserves to resume growth after resting stage and to develop reproductive organs in the next following season.

### 3.3. Leaf Traits

Salt stress often induces profound anatomical adjustments in leaves, which was also observed in this study. Under control conditions, DLBA developed leaves that were thicker, trichome-rich and characterized by a wide and loosely arranged spongy parenchyma. Such structure is commonly associated with enhanced evaporative buffering, higher internal CO_2_ diffusion capacity and an increased ability of the mesophyll to store and redistribute water [39,40]. In contrast, DH1 leaves were thinner, with scarce trichomes and a disproportionately thick palisade layer, resulting in a high palisade-to-spongy parenchyma ratio (6.7). This anatomy indicates that DH1 has a more light-oriented leaf architecture with a lower capacity for hydraulic buffering. These structural differences imply that DLBA possesses a constitutively more flexible and stress-ready leaf structure, whereas the rigid arrangement in DH1.

Under salt stress, the architecture of DH1 leaves shifted towards a pattern typical of salt-sensitive plants. The palisade to spongy parenchyma ratio substantially declined (from 6.7 to 1.8) due to the decrease in thickness of the palisade layer by 22% and almost 3-fold thickening of the spongy layer. The expansion of the spongy parenchyma occurred without increase in intercellular space, indicating tissue swelling rather than functional rearrangement that would improve internal CO_2_ conductance. In addition, the upper epidermis thickened by 37%, a response often associated with mechanical stress and the accumulation of osmotic solutes [41]. This combination of changes is consistent with structural disruption of the mesophyll under salt stress that is commonly correlated with reduced leaf hydration in salt-sensitive plants [22,40]. Together, the results indicate that DH1 leaves undergo structural destabilization under salt stress, with impaired mesophyll organization and reduced capacity to maintain efficient internal gas exchange.

In contrast, in DLBA, the upper epidermis thickened only moderately under salt stress, and the thickness of the palisade parenchyma remained unchanged, preserving its photosynthetic apparatus. The spongy parenchyma expanded by 56%, and unlike in DH1, this increase was accompanied by a substantial expansion of intercellular space (2.5-fold relative to DH1). Such a coordinated expansion is known to enhance internal gas diffusion and, secondarily, contribute to the mesophyll’s water-buffering capacity during stress [42]. Thus, DLBA maintains mesophyll integrity and functional leaf architecture under salt stress.

In many plant species, dense trichome cover is an adaptive feature associated with reduced leaf temperature, lower transpiration rate and the formation of a humid boundary layer around the epidermis [43,44]. In our study, DH1 produced almost no trichomes under control conditions, indicating a low inherent capacity for microclimate regulation at the leaf surface, whereas DLBA displayed high trichome density. The DH1 line is an orange cultivated carrot derived from a Western-type Nantes. It therefore shows a morphology typical of temperate-climate cultivars commonly grown in the USA and Europe, characterized by weak pubescence on both leaves and petioles. In carrot germplasm, Western-type accessions are known to exhibit markedly lower trichome density than Eastern-type materials, reflecting domestication-driven selection for smoother foliage and petioles. This background is consistent with the phenotype observed in DH1. The DLBA accession is an Eastern-type carrot, a type originating from hot and dry regions and characterized by typically pubescent leaves. Under salt stress, DLBA exhibited a directional adjustment in trichome distribution. The trichome density decreased on the adaxial surface but increased on the abaxial surface. Such a shift is consistent with adaptive responses reported in species exposed to water deficiency, where increased abaxial trichome density enhances the humidity around stomata and limits transpirational water loss. A lowered number of trichomes on the adaxial surface decreases light reflection and helps maintain photosynthetic capacity [45]. These modifications indicate that DLBA actively reorganizes its leaf protective structures under salt stress, while DH1 lacks this capacity due to its smooth leaves.

The above discussed findings complement root anatomical observations showing superior hydraulic safety in DLBA and suggest that tolerance to salinity in this accession results from coordinated structural resilience in both leaves and roots.

### 3.4. Chemical Composition of Cell Walls

In roots, galactan (LM5) and arabinan (LM6) pectic epitopes exhibited similar distribution patterns in both accessions regardless of the treatment, although a noticeable absence was observed in the xylem vessels under salt stress. In contrast, more pronounced constitutive differences were evident in the leaves. DLBA leaves contained galactans and arabinans in all tissues under both conditions, while in DH1 the occurrence of both epitopes was limited under control conditions and arabinans disappeared entirely under salt stress. β-1,4-galactan has been postulated to act as a water-retaining, viscoelastic component that modulates the mechanical properties of the cell wall [46,47,48] and arabinans function as plasticizers, enhancing cell wall elasticity and hydration under abiotic stress conditions [49,50,51,52]. Thus a stable presence of both side chains in DLBA leaves supports a more elastic and hydrated mesophyll. In DH1, the stress-induced loss of arabinans is consistent with reduced leaf wall flexibility and with the observed mesophyll destabilization as discussed in the previous section.

Highly methyl-esterified HG (LM20) was more abundant in leaves and roots of DH1 and DLBA under salt stress compared with controls (in particular in the salt-stressed DLBA roots where LM20 epitope was detected additionally in cortex), although the differences were estimated only by visual comparison of fluorescence intensity. A higher degree of pectin esterification has been associated with wall stiffening and turgor maintenance under stress conditions [53,54]. The salt-induced expansion of these pectins suggests a targeted reinforcement of cortical and xylem-related tissues. Together with anatomical data showing higher theoretical hydraulic safety in DLBA, this indicates that HG esterification is part of a coordinated protective strategy in this accession, whereas in DH1 similar changes are less pronounced. Low methyl-esterified HG (LM19) was absent in all tissues in both accessions, indicating that salinity responses in carrot rely mainly on changes in highly esterified HG and side-chain composition rather than changes in pectin esterification level.

Arabinogalactan proteins (AGPs) are actively involved in both abiotic and biotic stress responses. They play essential roles in mediating cell signaling, modulating cell wall architecture, and interacting with pathogens and environmental stressors (for review see [55]). Under stress conditions, their synthesis is dynamically reprogrammed, enabling plants to adapt to adverse environments. Few studies to date have described changes in AGPs under salt stress and showed either the increase or decrease in AGP levels, depending on the plant species and experimental conditions. Most of these studies concern AGP secretion into the medium during in vitro culture under salinity [55]. In general, the role of AGPs under salt stress is multifaceted, encompassing processes such activation of signaling pathways through the release of extracellular AGPs and cell wall stiffening associated with reduced AGP levels [55].

Immunohistochemical analyses showed that among the three AGP epitopes examined in the present study, only JIM13 was abundant in the cell walls, with no evident treatment-dependent variation either in roots or leaves. However, in DH1 salt stress-treated roots the JIM13 fluorescence signal was lost in vessel walls, while DLBA maintained it. This may suggest better preservation of AGP continuity at the junction between the vessel and parenchyma in DLBA roots. The JIM16 epitope exhibited a different occurrence pattern. Its signal decreased under salt stress in DH1 roots and was almost absent in DLBA in both conditions. It was also completely absent in leaves of both control and NaCl-treated DH1 and DLBA plants. AGPs, recognized by LM2 antibody, capable of binding Ca^2+^/Na^+^ [56,57], showed the strongest accession specificity. LM2 epitope was present in many tissues of DH1 roots and leaves, regardless of treatment. DLBA roots had no LM2 signal at all while in DLBA leaves it was detected only in vascular bundles under salt stress. This suggests that DLBA may use AGPs, that are detected by LM2 antibody, in a restricted and stress-dependent manner to most probably manage ion movement locally, unlike DH1 which deploys them broadly and in a constitutive manner.

Previous research on miRNA expression in DH1 and DLBA demonstrated upregulation of the *arabinogalactan protein 9*-like (*agp9l*) gene in the roots of salt-treated DLBA plants [16]. Thus gene expression and immunolocalization results for AGPs may appear inconsistent. However, JIM13, JIM16, and LM2 detect carbohydrate epitopes, not AGP backbones [56], so immunodetection does not necessarily reflect expression of specific AGP genes such as *agp9l*.

Hemicelluloses are complex group of glycans that constitute a major component of the plant cell wall. They strengthen the cellulose network and provide mechanical support. Plants exposed to salt stress exhibited higher levels of xylose compared with controls [54]. As previously reported, the increased xylose level under salt stress indicates that the xylan backbone displays a lower degree of substitution, which contributes to cell wall stiffening and allows cells to better resist internal turgor pressure [54,58,59]. 

In the present study, a higher content of xylans and xyloglucans (detected using the LM10 and LM25 antibody, respectively) was also observed in DH1 and DLBA roots under salt stress, compared with control, particularly in xylem parenchyma cells of DLBA. These changes in the hemicellulose composition of cell walls in salt-stressed roots may therefore explain a postulated safer hydraulic architecture in DLBA. 

In the leaves, the distribution of hemicelluloses showed clearer differentiation between accessions. In DH1 under control conditions, xyloglucan occurred in both the upper and lower epidermis, mesophyll cells and vascular tissue. Under salt stress in DH1, fluorescence signal was restricted to epidermal cells, indicating a reduction in hemicellulose reinforcement in mesophyll and vascular bundles. Such loss can weaken wall rigidity and contribute to the structural destabilization observed in DH1 leaves. In contrast, DLBA displayed a simpler but more stable pattern of LM10 and LM25 epitope occurrence. Xylans were confined to vascular bundles and xyloglucans to the epidermis, and this pattern remained unchanged under salt stress. This stability is consistent with the preserved mesophyll organization observed in DLBA leaves. These differences among accessions and treatments are also congruent with earlier gene expression data showing that in the leaves, the expression of *xyloglucan 6-xylosyltransferase 1*-like (*xxt1*) gene, involved in xyloglucan biosynthesis, was significantly lower in DLBA than in DH1, and decreased under salt stress in both accessions [16].

Extensins are structural cell wall glycoproteins (HRGPs). During cell differentiation, extensins accumulate in the primary wall and crosslink the cellulosic framework, forming a rigid and inextensible matrix [60]. The formation of such crosslinks strengthens the wall, helping cells withstand osmotic fluctuations induced by salt stress [50]. 

The results presented here show that under salt stress, extensin levels increased in the roots of both accessions. In contrast, this epitope disappeared in DH1 leaves and was absent in DLBA leaves in either treatment.

Extensins (recognized by JIM20 antibody) strengthened root vessel walls in both accessions and expanded modestly into xylem parenchyma under salinity, suggesting a general protective response [50,60]. In leaves, however, JIM20 patterns were accession-specific: DH1 lost extensin signal from vascular bundles under salinity, whereas DLBA showed no detectable JIM20 epitope in either treatment. The loss of extensin in DH1 is analogous as in *Nicotiana tabacum* where a reduced extensin–cellulose matrix weakened wall tensile strength [60], and consistent with the DH1 mesophyll collapse under stress. The complete absence of the JIM20 epitope in DLBA leaves remains unexplained. It is possible that DLBA leaves contain extensins recognized by other antibodies, such as JIM11, LM1, JIM12, or JIM19, which were not used in this study.

### 3.5. Tolerance Strategies to Salt Stress

The revealed histological and immunohistological findings show that the two *Daucus carota* accessions rely on different strategies to cope with soil salinity. A salt stress-tolerant DLBA exhibits a set of constitutive structural traits such as thicker leaves with dense trichomes, elastic and hydrated walls enriched in galactans and arabinans, stable patterns of hemicellulose occurrence, and a hydraulically safe root xylem. Under control conditions they provide mechanical flexibility, water-buffering capacity and protection of essential tissues. Under salt stress, DLBA has several ongoing adaptive processes. Highly esterified HG expands in root cortical and xylem parenchyma cell walls, hemicellulose deposition increases around the vascular system, and AGPs deployment is spatially restricted. This integrated pattern suggests a pre-adapted wall organization capable of stabilizing tissue hydration, preserving gas diffusion and supporting continuous water transport.

In contrast, DH1 shows traits characteristic of a salt-sensitive phenotype. Its leaves are rigid, with limited wall elasticity and almost no trichomes. Its roots possess fewer vessels and lower hydraulic redundancy. Under salt stress, DH1 undergoes largely uncoordinated changes. These include strong shifts in palisade to spongy parenchyma proportions, loss of arabinan epitopes, reduction in hemicellulose reinforcement in leaves, disappearance of extensin from vascular bundles, and disrupted AGP continuity in roots. Combined with reduced vessel number and diameter, these changes point to a destabilized hydraulic system and diminished capacity to maintain functional wall mechanics under osmotic stress.

The present findings align with earlier physiological studies on the same accessions [14,15,16], which showed higher productivity, stronger antioxidant responses, lower oxidative damage, more stable carbohydrate balance and more efficient mechanisms maintaining ion homeostasis in the salt-tolerant DLBA than in the salt-sensitive DH1 plants. The current evidence at the anatomical, histological and cell wall composition levels expands these earlier conclusions by demonstrating that the salt tolerance of DLBA plants arises from a combination of favorable features related to leaf architecture, starch accumulation, xylem organization and cell wall remodeling. These results provide a structural and mechanistic basis for previously documented physiological processes, showing that DLBA plants are not only more responsive to salt stress but also have inherent structural traits that enhance resilience. Thus, the findings integrate physiological and structural perspectives, offering a more complete understanding of how DLBA plants maintain tissue integrity and functionality under salt stress.

## 4. Materials and Methods

### 4.1. Plant Material

Carrot (*Daucus carota* L. ssp. *sativus* Hoffm.) plants were subjected to salt stress treatment as described previously [14]. In brief, two accessions were used: the salt-tolerant DLBA and the salt-sensitive DH1 (obtained from Prof. P.W. Simon, University of Wisconsin-Madison and USDA-ARS, Madison, WI, USA). The accessions are deposited in the carrot collection at the University of Agriculture in Krakow, Poland. Plants were growing in plastic containers filled with soil of EC = 3.0 dS·m^−1^ (salt stress). Soil salinity was maintained by irrigation with a 100 mM NaCl solution during the vegetation period. The control plants were grown in soil of EC = 0.2 dS·m^−1^. Root and leaf samples were collected from 15-week-old plants. 

### 4.2. Bright Field Microscopy

Root discs were excised from the central part of the developed storage roots and prepared according to the procedure described earlier [61]. Briefly, samples were fixed in a mixture of 3% (*w*/*v*) paraformaldehyde (PFA) (Polysciences, Warrington, PA, USA) and 1.25% (*v*/*v*) glutaraldehyde (GA) (Sigma-Aldrich, St. Louis, MO, USA) in phosphate-buffered saline (PBS, pH = 7.2) at 4 °C overnight, then washed, dehydrated through an increasing ethanol series (10, 30, 50, 70, 90 and 100%; *v*/*v*; two times, 30 min each), and embedded in Steedman’s wax. Sections 8 µm thick were cut using a Zeiss HYRAX M40 rotary microtome (Jena, Germany) and mounted on microscopic slides coated with Mayer’s albumin or poly-L-lysine (Menzel Gläser, Braunschweig, Germany).

Leaf blade samples were taken from fully developed leaves, specifically the third youngest leaf in the rosette, and processed according to the procedure described by Milewska-Hendel et al. [62]. Briefly, samples were fixed in a mixture of 4% PFA and 1% GA in PBS (pH = 7.2), overnight at 4 °C, then dehydrated through a graded ethanol series and gradually embedded in LR White resin (Polysciences, Warrington, PA, USA). After polymerization, the samples were cut into 1.5 µm sections using an EM UC6 ultramicrotome (Leica Biosystems, Zalesie Gorne, Poland). 

For histological analyses, sections were stained with 0.1% toluidine blue O (TBO; Sigma-Aldrich, St. Louis, MO, USA) in PBS (pH = 7.3) and examined under an Olympus BX45 microscope (Olympus, Tokio, Japan) equipped with an Olympus XC50 digital camera (Olympus, Tokio, Japan).

### 4.3. Immunohistology

For the immunofluorescence staining, sections were outlined with a hydrophobic marker (PAP pen; Sigma-Aldrich) and submerged in a blocking buffer containing 2% (*v*/*v*) fetal calf serum (FCS; Sigma-Aldrich, St. Louis, MO, USA) and 2% (*w*/*v*) bovine serum albumin (BSA; Jackson ImmunoResearch Laboratories, West Grove, PA, USA) in PBS for 30 min. Primary monoclonal antibodies (Plant Probes, Leeds, UK) were diluted 1:20 in blocking buffer and applied to the sections (overnight at 4 °C). The antibodies used are listed in Table 3. After three washes in blocking buffer, sections were incubated for 2 h with the secondary antibody (Alexa Fluor 488 goat anti-rat IgG, Jackson ImmunoResearch Laboratories, West Grove, PA, USA), diluted 1:100 in the blocking buffer. After three washes in blocking buffer and three washes in PBS, sections were stained with 0.01% (*w*/*v*) Fluorescent Brightener 28 (FB28; Sigma-Aldrich, St. Louis, MO, USA) in PBS for 5 min. Finally, after three washes in PBS and three in ultra-pure water, sections were mounted in Fluoromount (Sigma-Aldrich, St. Louis, MO, USA) anti-fading medium. Negative controls were prepared by omitting the primary antibody. Sections were analyzed using a Nikon Eclipse Ni-U microscope equipped with a Nikon Digital DS-Fi1-U3 camera and corresponding software (Nikon, Tokyo, Japan) at excitation wavelengths of 450–490 nm (AlexaFluor 488) and 330–380 nm (Fluorescent Brightener 28). All images were captured under the same acquisition conditions, with constant fluorescent lamp intensity, exposure time, and imaging parameters across all samples. Epifluorescence microscopy images were prepared as figures using Corel Draw 22.0 (2020) and Corel Photo-Paint 22.0 (2020) software, with brightness and contrast adjusted as needed.

### 4.4. Statistical Analysis

Cambial cells were counted in 50 and vessels in 10 randomly chosen radial rows per root section. Vessel diameter was measured for 50 vessels, and starch grains were counted in 100 cells. Leaf characteristics were determined by performing 50–100 measurements of leaf cross-sections. Observations were performed for three to five plants in each treatment. A two-factor ANOVA with interaction was applied to test the effects of accession and treatment. The Newman–Keuls multiple comparison test was used to determine significant differences between means at the 0.05 significance level.

## 5. Conclusions

The presented results demonstrate that the salt-tolerant carrot DLBA plants possess both constitutive and stress-induced traits that stabilize water transport and preserve mesophyll organization under salt stress. In contrast, the salt-sensitive DH1 plants exhibit a rigid leaf architecture and weaker xylem safety, leading to structural destabilization in leaves and reduced hydraulic capacity in roots. Differences in the distribution of pectins, hemicelluloses, AGPs and extensins indicate that DLBA accession maintains a coherent wall-based strategy for maintaining hydration, elasticity and vascular integrity, whereas DH1 shows stress-driven disruption of these processes. Together, these findings highlight that salinity tolerance in carrot is strongly linked to an integrated anatomical and cell-wall resilience framework rather than to isolated biochemical adjustments.

## Figures and Tables

**Table 1:** Quantitative anatomical traits of root cross-sections in DH1 and DLBA carrot accessions under control and salt stress conditions.

	DH1		DLBA	
Anatomical Characteristics	Control	NaCl	Control	NaCl
Mean number of cambial cells	4.1 (1.07) c	2.6 (0.6) d	6.5 (1.2) a	5.3 (0.8) b
Mean vessel diameter (µm) (d)	31.6 (11.3) a	25.5 (8.3) b	34.3 (6.3) a	27.3 (6.3) b
Percentage of radial rows with vessels (%) (R)	53.3 (11.5) a	50.0 (10.0) a	43.3 (30.6) a	73.3 (11.5) a
Percentage of vessels per radial row (%) (V)	16.7 (18.8) b	9.7 (11.8) b	8.3 (11.7) b	24.4 (16.8) a

Values in parentheses are standard deviations. Different letters indicate significant differences at *p* < 0.05 (Newman–Keuls test).

**Table 2:** Anatomical traits of the DH1 and DLBA leaf blades under the control and salt stress conditions.

	DH1						DLBA					
Leaf Blade Trait	Control			NaCl			Control			NaCl		
Thickness (µm)												
leaf blade	264.4	(13.0)	c	289.3	(24.4)	bc	306.3	(30.8)	ab	357.5	(17.9)	a
upper epidermis	32.4	(0.8)	b	44.3	(1.2)	a	35.2	(2.9)	b	41.0	(4.6)	a
palisade parenchyma	177.6	(16.4)	a	138.1	(20.8)	b	127.1	(16.2)	b	119.5	(9.5)	b
spongy parenchyma	26.5	(8.6)	c	76.7	(12.2)	b	105.7	(44.2)	b	165.3	(22.9)	a
lower epidermis	27.9	(2.1)	a	30.2	(2.7)	a	27.1	(2.2)	a	31.7	(4.5)	a
Air spaces in spongy parenchyma (%)	7.3	(2.1)	b	5.5	(2.3)	b	13.1	(2.8)	a	13.6	(3.4)	a
Trichomes (No./mm^2^)												
upper epidermis	nd			nd			33.6	(4.5)	a	19.1	(2.7)	b
lower epidermis	nd			nd			15.1	(2.8)	b	23.2	(1.9)	a

Values are means followed by standard deviations in parentheses. Different letters indicate significant differences at *p* < 0.05 (Newman–Keuls test); nd—not determined.

**Table 3:** Primary antibodies used for the detection of cell wall components. AGPs—arabinogalactan proteins, GalA—galacturonic acid, GlcA—glucuronic acid, HG—homogalacturonan, HRGP—hydroxyproline-rich glycoproteins, RG I—rhamnogalacturonan I, Rha—rhamnose.

Component	Antibody	Epitope	References
Pectins	LM5	Linear tetrasaccharide in (1 → 4)-β-D-galactans (RG I side chain)	[63]
	LM6	Arabinan (RG I side chain)/(1,5)-α-L-arabinan (also labels AGPs)	[64]
	LM19	Unmethyl-esterified, partially methyl-esterified HG	[65]
	LM20	Methyl-esterified HG	[65]
AGP	JIM13	β-D-GlcA-(1 → 3)-α-D-GalA-(1 → 2)-L-Rha	[66]
	LM2	Arabinogalactan/arabinogalactan protein, carbohydrate epitope containing β → linked GlcA	[67]
	JIM16	Arabinogalactan, Arabinogalactan protein	[66]
Extensin	JIM20	Extensin/HRGPs	[68]
Hemicelluloses	LM10	Specific to unsubstituted or low-substituted xylans	[69]
	LM25	xylosylated/galactosylated oligosaccharide motifs of xyloglucan	[70]

## Data Availability

The original contributions presented in the study are included in the article. Further inquiries can be directed to the corresponding author.
